# Correlation between carpal rotational alignment and postoperative wrist range of motion following total wrist arthroplasty

**DOI:** 10.1186/s12891-022-05776-x

**Published:** 2022-08-30

**Authors:** Mitsutoshi Ota, Yuichiro Matsui, Daisuke Kawamura, Atsushi Urita, Takeshi Endo, Norimasa Iwasaki

**Affiliations:** 1 Department of Orthopaedic Surgery, Obihiro Kosei Hospital Hand Center, Obihiro, Japan; 2 Faculty of Dental Medicine, Hokkaido University, N13 W7, Kita-Ku, Sapporo, Hokkaido 060-8586 Japan; 3 Department of Orthopaedic Surgery, Faculty of Medicine and Graduate School of Medicine, Hokkaido University, Sapporo, Japan; 4 Department of Orthopaedic Surgery, Dokkyo Medical University, Mibu, Japan

**Keywords:** Range of motion, Rheumatoid arthritis, Total wrist arthroplasty, Wrist alignment

## Abstract

**Background:**

Although total wrist arthroplasty (TWA) has become a common treatment option for wrists with damage due to rheumatoid arthritis (RA), the optimal implant axial alignment for TWA has been inadequately studied. This study was performed to investigate the relationships between implant alignment and carpal rotational alignment and the wrist range of motion (ROM) following TWA.

**Methods:**

This study included 18 patients who underwent TWA using a DARTS® Total Wrist System (Teijin Nakashima Medical, Okayama, Japan) for wrist RA. Pre- and 6-month postoperative computed tomography scans were performed, including the radial volar line (Rv), capitohamate axis (CH), and Rv-CH angle in axial scans. The wrist ROM was also measured. The relationship between the Rv-CH angle and ROM was examined.

**Results:**

The mean Rv-CH angle showed significant wrist pronation from 73.0° to 83.4° postoperatively. We observed a significant positive correlation (0.58) between the postoperative Rv-CH angle and extension and a significant negative correlation (− 0.56) between the postoperative Rv-CH angle and flexion.

**Conclusions:**

Implantation of the DARTS® TWA prosthesis resulted in pronation of the carpal axial alignment, which was correlated with postoperative wrist extension. The volar cortex of the distal radius can be a novel reference axis for adequate implant placement.

## Background

**Fig. 1:**
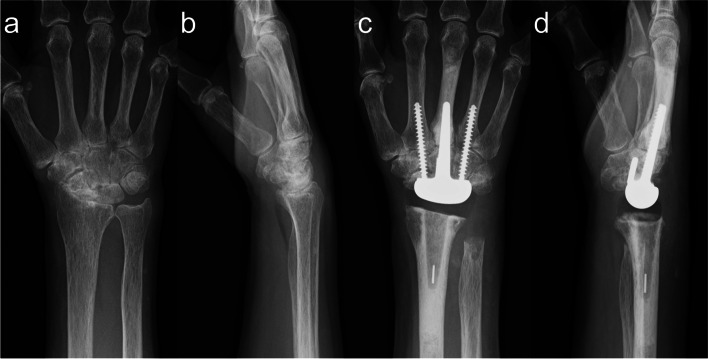
Preoperative (**a**) posteroanterior and (**b**) lateral radiographs of the right wrist of a 66-year-old woman with RA. **c** Posteroanterior and (**d**) lateral radiographs at 6 months after DARTS® TWA

Total wrist arthroplasty (TWA) can be a successful treatment option for wrists destroyed by rheumatoid arthritis (RA) or osteoarthritis because it allows preservation of the range of motion (ROM) [1]. Modern implants such as the Universal 2™ (Integra LifeSciences, Princeton, NJ, USA), ReMotion™ (Stryker, Kalamazoo, MI, USA), and Maestro™ (Zimmer Biomet, Warsaw, IN, USA) reportedly improve patient-related outcome measures and pain scores [2, 3]. We previously developed the DARTS® Total Wrist System (Teijin Nakashima Medical, Okayama, Japan) and reported its favourable clinical outcomes [3] (Fig. 1).

The placement alignment of total knee arthroplasty (TKA) has been extensively studied, and the rotation alignment at the time of placement has been shown to be associated with the clinical results [4–7]. Studies have also been conducted on anatomical baselines that can be used as indicators of proper implant placement [6]. Biomechanical investigations are important to understand the wrist biomechanics after TWA, especially the possible causes of mechanical failure of this procedure [8, 9]. TWA has a history as long as that of TKA [10]. However, the optimal implant rotation angle for TWA has not been clarified.

**Fig. 2:**
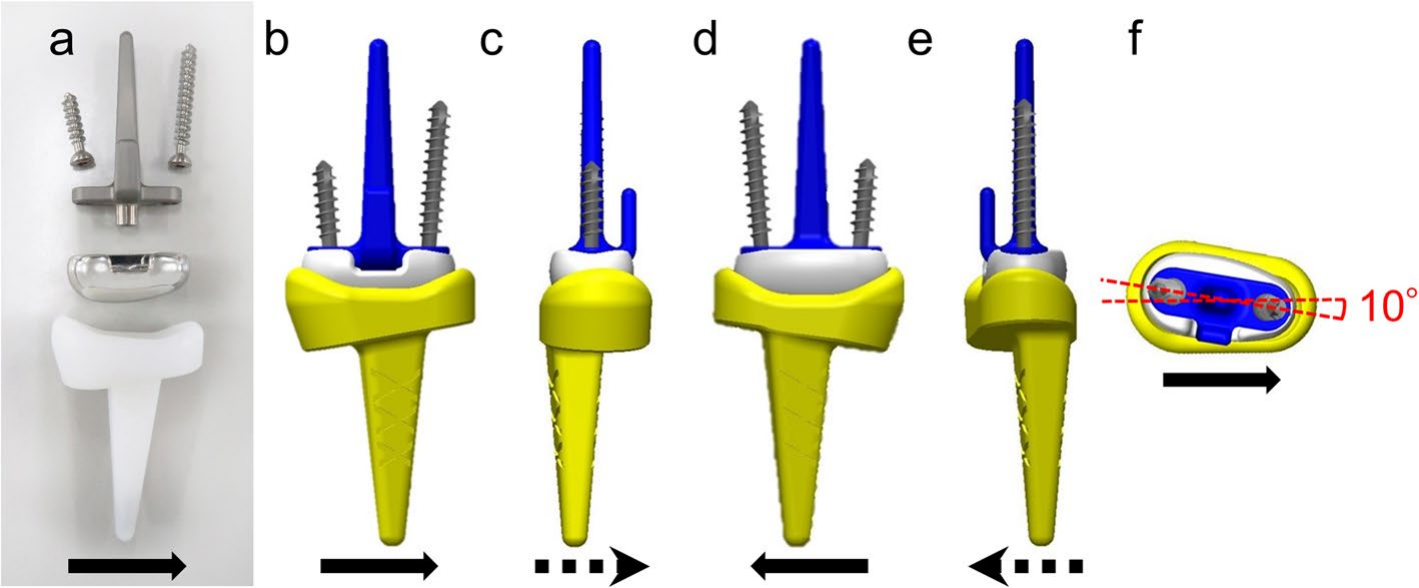
**a** Frontal photograph of DARTS® prosthesis for a right wrist. **b** Frontal view, **c** view from ulnar side, **d** posterior view, **e** view from radial side, and **f** top-down view of the three-dimensional model. Solid arrows indicate the radial side, and dashed arrows indicate the volar side

The DARTS® prosthesis consists of radial and carpal components (Fig. 2a–e). One of the defining features of the DARTS® prosthesis is that the flexion–extension axis is pronated by 10° around the line of intersection of the axial plane [3] (Fig. 2f). The prosthesis is designed such that this rotation will allow the dart-thrower’s motion, minimising the load on the soft tissue around the wrist [11, 12]. However, the relationship between the actual postoperative carpal alignment and ROM in patients is not well understood.

We hypothesised that the DARTS® prosthesis improves wrist ROM by changing the carpal rotational alignment. This study was performed to examine the relationships between implant alignment and carpal rotational alignment and the postoperative wrist ROM with the DARTS® prosthesis. This study also provides an anatomical reference axis as an indicator of adequate implant placement.

## Methods

### Participants

After receiving approval from the institutional review board, this study included all 18 patients (mean age, 64.3 ± 8.8 years; 2 men, 16 women) with RA who underwent TWA using the DARTS® Total Wrist System from February 2010 through March 2020. All patients had severely damaged wrist joints, defined as Larsen grade [13] IV to V. The procedures were performed by expert hand surgeons [14].

### Design of the prosthesis and surgical technique

The semiconstrained prosthesis consists of ultra-high molecular weight polyethylene (UHMWPE) radial and titanium-6alminum-4 vanadium (Ti-6Al-4 V) carpal components, Ti-6Al-4 V bone screws, and a cobalt-chromium-molybdenum carpal head [3]. The radial component and the central stem of the carpal component are fixed with bone cement. At the time of its development, the prosthesis was designed for patients with poor osteoporotic or rheumatoid bone stock. Some surgeons recommend to implant prostheses without cement because of the advantage of stability achieved through osseous integration, maintenance of bone stock, and theoretical longevity [15, 16]. The details of the surgical procedure have been described previously [3]. Under general anaesthesia, a skin incision was made from the third metacarpal base, proximal to Lister’s tubercle. The extensor retinaculum was then opened from the first to fifth compartments, and the dorsal wrist capsule was raised. The distal parts of the ulna and radius were resected. After rasping the medullary cavity of the radius, the implant size was determined. The proximal part of the capitate was resected, and a drill was introduced to the capitate and third metacarpal. After rasping the capitate and third metacarpal, the implant size was determined. The radial component and the central stem of the carpal component were inserted and fixed with bone cement (Endurance®; DePuy Synthes, Inc., Warsaw, IN, USA). The carpal component was further secured by two screws into the second and fourth metacarpals, and the radial component and carpal head were articulated. The distal ulna was stabilised using a proximally based half-slip of the extensor carpi ulnaris [17, 18].

### Computed tomography image acquisition

Pre- and 6-month postoperative transverse computed tomography (CT) scans (Aquilion ONE™ ViSION; Canon Medical Systems, Ōtawara, Japan) of the entire wrist were performed. All CT data were sourced from a digital image archive system (Picture Archiving and Communications Systems [PACS] Synapse; Fujifilm Inc., Tokyo, Japan), and all measurements were performed using the PACS. The following marks and measurements were made based on the CT axial scans.

**Fig. 3:**
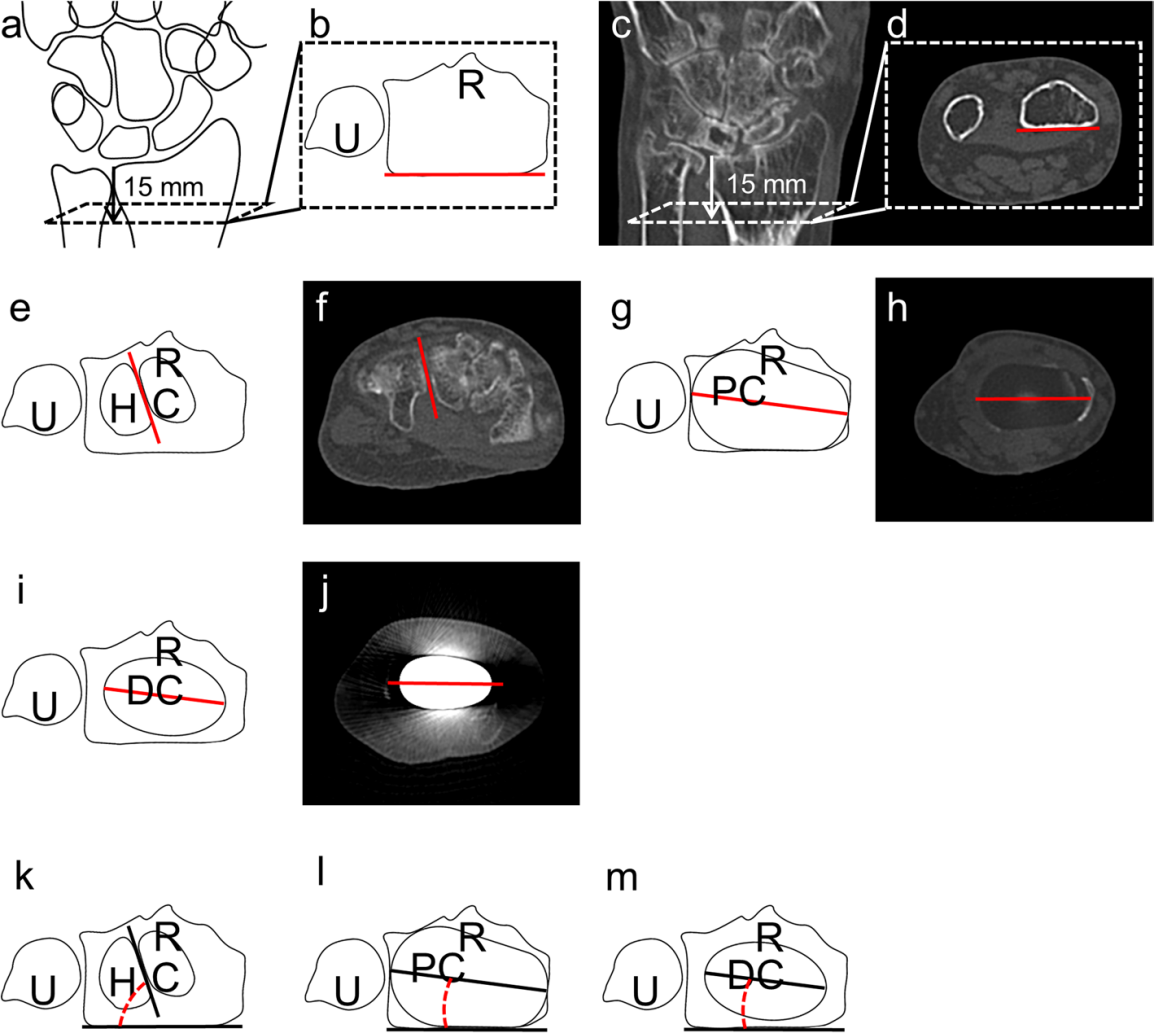
Diagrammatic representations and typical CT images of various axes and angles. **a**–**d** Radial volar line (Rv), defined as the volar cortex of the distal radius 15 mm proximal to the ulnar edge of the distal radioulnar joint surface. **e** and **f** Capitohamate axis (CH). **g** and **h** Proximal component axis (PC). **i** and **j** Distal component axis (DC). **k** Rv-CH angle. **l** Rv-PC angle. **m** Rv-DC angle. R: radius; U: ulna; C: capitate; H: hamate; PC: proximal component; DC: distal component

1. The radial volar line (Rv) was defined as the line connecting the radial and ulnar edges of the volar cortex of the distal radius 15 mm proximal to the ulnar edge of the distal radioulnar joint surface (Fig. 3a–d). The same line was identified after surgery based on the measurement of the length before surgery.
2. The capitohamate axis (CH) was defined as a line parallel to the capitohamate joint (Fig. 3e, f).
3. The proximal component axis (PC) was defined as the centre of the proximal component (Fig. 3g, h).
4. The distal component axis (DC) was defined as the axis connecting the distal fixation screw heads (Fig. 3i, j).
5. The angle between the Rv and the CH was defined as the Rv-CH angle (Fig. 3k).
6. The angle between the Rv and the PC was defined as the Rv-PC angle (Fig. 3l).
7. The angle between the Rv and the DC was defined as the Rv-DC angle (Fig. 3m).

The CT scans were performed with the patients sitting and keeping the shoulder horizontally abducted at 90° with the elbow flexed at 90° and the palm facing down flat on the table. In all cases, the capitohamate articular surface was maintained and measurements were possible.

After 4 weeks, the patients’ CT scans were measured again by the same author, while another author conducted an independent evaluation to determine the intraobserver and interobserver variabilities to determine the intraclass correlation coefficients (ICCs), with values of > 0.8 indicating excellent agreement, 0.6 to 0.8 indicating fair to good agreement, and < 0.6 indicating poor agreement. Both observers were blinded to the ROM measurements.

### Wrist ROM measurement

Flexion, extension, radial deviation, ulnar deviation, pronation, and supination were measured before and 6 months after surgery.

### Statistics

The relationships between the angles (Rv-CH, Rv-PC, and Rv-DC angles) and ROM were compared. Statistical analysis software (JMP® Pro version 16.0; SAS Institute, Cary, NC, USA) was used for data processing for analysis of variance with appropriate post hoc multiple comparisons, including non-parametric methods when applicable. Spearman’s correlation coefficient was used to measure the relationship between two related variables. ICCs were calculated according to standard statistical methods (ICC 1,2 for intra-rater; ICC 2,1 for inter-rater). The ICCs were classified as slight (< 0.20), fair (0.21–0.40), moderate (0.41–0.60), substantial (0.61–0.80), or almost perfect agreement (0.81–1.00) [19]. The level of significance was set at *P* < 0.05.

## Results

**Fig. 4:**
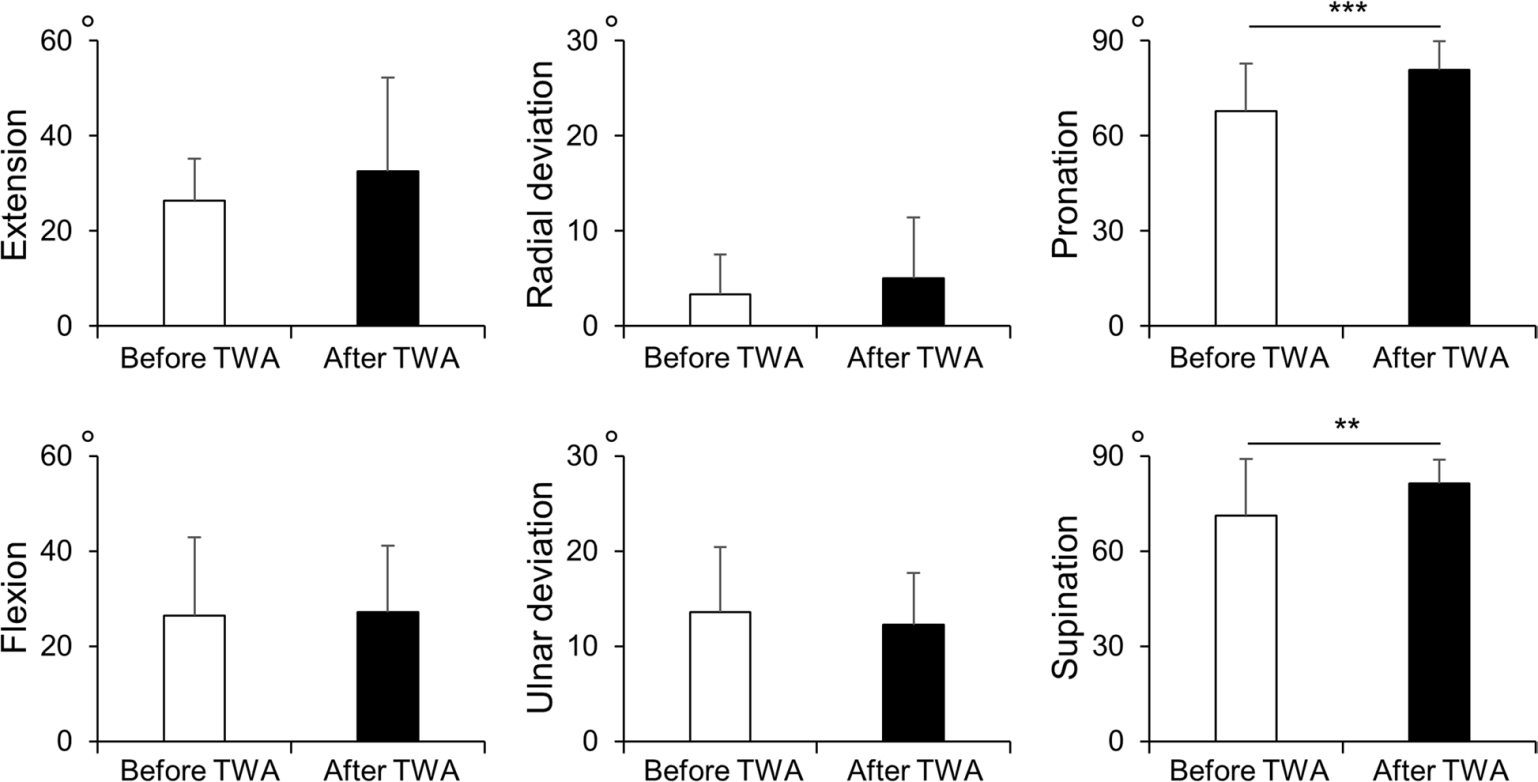
Wrist ROM before and after TWA. **P < 0.01, ***P < 0.001

**Fig. 5:**
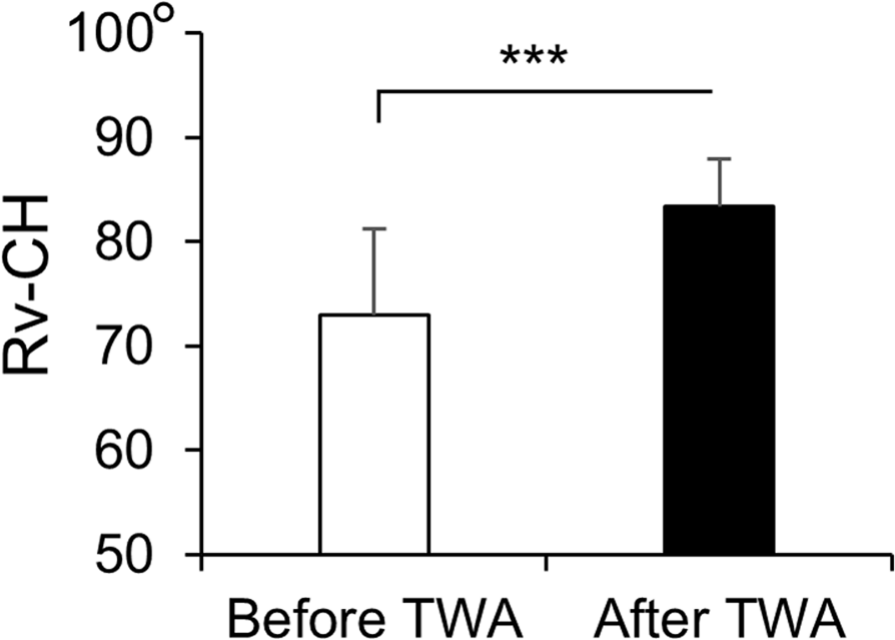
Mean Rv-CH angles before and after TWA. ***P < 0.001

The ICCs for the reproducibility of all parameters showed almost perfect agreement (> 0.81). The Rv-PC angle, Rv-DC angle, and Rv-CH angle were measured with Rv as the reference line. The mean Rv-PC and Rv-DC angles were 12.8° ± 8.9° and 14.1° ± 10.1°, respectively, with an ICC of 0.98 (*P* < 0.001). The mean wrist extension angle was 26.4° ± 8.9° to 32.5° ± 19.7° postoperatively, and the mean wrist flexion angle was 26.4° ± 16.4° to 27.2° ± 13.9° postoperatively. The mean wrist radial deviation angle was 3.3° ± 4.2° to 5.0° ± 6.4° postoperatively, and the mean wrist ulnar deviation angle was 13.6° ± 6.8° to 12.2° ± 5.5° postoperatively. The mean wrist pronation angle increased significantly from 67.8° ± 15.1° to 80.8° ± 9.0° postoperatively, and the mean wrist supination increased significantly from 71.1° ± 17.9° to 81.4° ± 7.4° postoperatively. The ROM before and after TWA are shown in Fig. 4. The mean Rv-CH angle was significantly pronated from 73.0° ± 8.2° to 83.4° ± 4.6° postoperatively (Fig. 5).

**Fig. 6:**
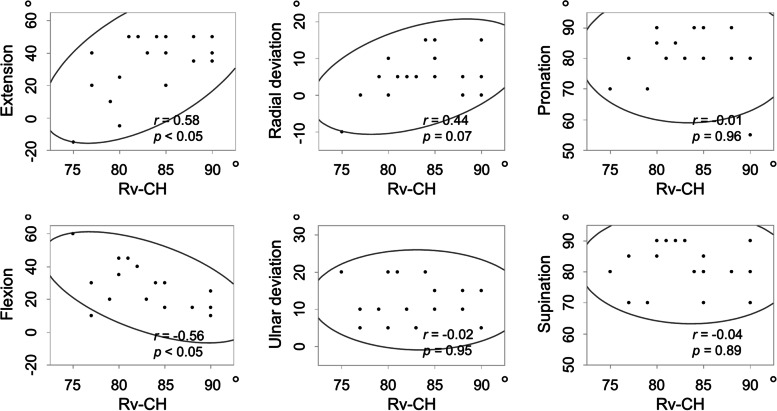
Correlation between Rv-CH angle and postoperative wrist range of motion

A significant positive correlation was observed between the postoperative Rv-CH angle and postoperative wrist extension (Fig. 6). Furthermore, a significant negative correlation was observed between the postoperative Rv-CH angle and postoperative wrist flexion (Fig. 6). In contrast, no significant correlation was found between the postoperative Rv-CH angle and other postoperative wrist ROM parameters, such as radial deviation, ulnar deviation, pronation, and supination. During the observation period in this study, there were no obvious complications such as loosening of the implant or infection, and no reoperation was necessary.

## Discussion

TWA has greatly improved since it was first reported by Swanson [20] in 1973. Whereas previous implants were associated with high rates of reoperation and complications, current implants have shown promising results in reducing these problems [21]. TWA shows good clinical outcomes and is a viable surgical option for the treatment of wrists with severe RA [1–3]. It is widely known that the rotational position of the components of TKA is important [4, 6, 7] and that rotational alignment is related to the ROM [5]. However, there are no reports on the relationship between the rotational alignment of the TWA and ROM. The present study investigated the relationship between the implant, carpal rotational alignment, and postoperative wrist ROM following TWA and provided an anatomical reference axis as an indicator of adequate implant placement. The main findings were a positive correlation between the pronation angle of the carpal alignment and the extension angle of the wrist and a negative correlation between the pronation angle of carpal alignment and the flexion angle of the wrist after DARTS® TWA.

The wrist ROM parameters, including extension, flexion, radial deviation, and ulnar deviation, did not change significantly postoperatively. These results indicate that the wrist ROM is preserved postoperatively, consistent with our previous report on DARTS® TWA [3]. However, the forearm ROM in pronation and supination was significantly improved postoperatively. These results are considered to be mainly due to the resection of the ulnar head [22].

The shape of the volar surface of the distal radius is concave in the axial plane at the radiocarpal joint surface and gradually flattens toward the proximal radius. The distance from the joint surface to the point where the volar surface of the distal radius turns flat is 16 mm [23]. This indicates that the Rv position, which we defined as the reference line, is appropriate in terms of ease of measurement and high reproducibility. Biomechanical analysis showed that the dart-thrower’s motion of wrist extension radial deviation and wrist flexion ulnar deviation minimises the tension of the ligaments and tendons around the wrist [11, 12]. Furthermore, this motion is necessary for a variety of daily tasks and may even provide a unique survival advantage for humans [24]. Crisco et al. [11] stated that a wrist implant should consider not only the anatomical axes but also the mechanical axes of wrist motion. The DARTS® total wrist prosthesis, which we developed and used in this study, is designed such that the flexion–extension axis is pronated 10° to imitate the dart-thrower’s motion [3]. This pronation is consistent with the physiological rotation of the distal radial articular surface [23].

To our knowledge, this is the first study to investigate the rotational alignment of TWA components. We showed that the DARTS® prosthesis deviated the flexion–extension axis by 10°, as designed. Our results revealed a positive correlation between the pronation of the carpal axial alignment and wrist extension and a negative correlation with wrist flexion. The patients’ mean postoperative ROM was 27.2° for wrist flexion and 32.5° for wrist extension. In our previous report [3], we did not discuss the respective angles of wrist flexion and extension. Based on the results of this study, our model, like other models [25], increases the wrist extension angle but not the flexion angle. This is a problem common to other models that has not been improved in our model. There are two possible causes of an impaired flexion angle. First, no types of prostheses, including our new prosthesis, restore the anatomical predetermined volar tilt of the distal radial articular surface in the sagittal plane [26]. Second, scarring around the surgical incision at the wrist capsule associated with the loss of its elasticity can be associated with impaired movement in the opposite direction after TWA [27]. Nevertheless, these ROMs in many cases satisfied the functional wrist ROM (5° of flexion and 30° of extension) reported by Palmer et al. [28]. Based on these results (Figs. 2, 3, 5, and 6), the volar ridge of the proximal component of the DARTS® should be parallel to the volar cortex of the distal radius at 15 mm proximal to the ulnar edge of the distal radioulnar joint surface to obtain functional wrist motion. A total wrist prosthesis design should reproduce the dart-thrower’s motion [8], and the DARTS® prosthesis may meet this criterion. Furthermore, the results of this study may be useful for other total wrist implants.

The present study has several limitations. First, the analysis included a relatively small number of patients. Second, the position of the arm during CT scanning might not have been consistent. Third, the relationship between the osteotomy volume and wrist ROM was not evaluated. Fourth, the relationship between carpal rotational alignment and ROM was examined for only 6 months after TWA. It is important to note that problems associated with unsuccessful TWA, especially polyethylene wear on the insert surface [29], begin at 5–8 years postoperatively [30]. Thus, longer follow-up is needed for the DARTS® prosthesis.

Despite these limitations, our results provide a further understanding of TWA and define a novel reference axis, the volar cortex of the distal radius, for implantation. A major finding is that the pronation of carpal rotational alignment was correlated with postoperative wrist extension. Future studies should include other total wrist prostheses to determine the relationship between carpal rotational alignment and wrist ROM.

## Conclusion

There is a positive correlation between the pronation of the carpal rotational alignment and the extension angle of the wrist following DARTS® TWA.

## Data Availability

The datasets used and/or analysed during the current study are available from the corresponding author on reasonable request.

## Abbreviations

CH: Capitohamate axis
CT: Computed tomography
DC: Distal component axis
ICC: Intraclass correlation coefficient
PC: Proximal component axis
Rv: Radial volar line
ROM: Range of motion
RA: Rheumatoid arthritis
Ti-6Al-4 V: Titanium-6alminum-4 vanadium
TKA: Total knee arthroplasty
TWA: Total wrist arthroplasty
UHMWPE: Ultra-high molecular weight polyethylene
